# Genetic Approaches for the Treatment of Facioscapulohumeral Muscular Dystrophy

**DOI:** 10.3389/fphar.2021.642858

**Published:** 2021-03-12

**Authors:** Kenji Rowel Q. Lim, Toshifumi Yokota

**Affiliations:** ^1^ Department of Medical Genetics, Faculty of Medicine and Dentistry, University of Alberta, Edmonton, AB, Canada; ^2^ The Friends of Garrett Cumming Research and Muscular Dystrophy Canada, HM Toupin Neurological Science Research Chair, Edmonton, AB, Canada

**Keywords:** facioscapulohumeral muscular dystrophy, DUX4, antisense oligonucleotides, RNAi, DNA decoys, DNA aptamers, CRISPR, gene modulation

## Abstract

Facioscapulohumeral muscular dystrophy (FSHD) is an autosomal dominant disorder characterized by progressive, asymmetric muscle weakness at the face, shoulders, and upper limbs, which spreads to the lower body with age. It is the third most common inherited muscular disorder worldwide. Around 20% of patients are wheelchair-bound, and some present with extramuscular manifestations. FSHD is caused by aberrant expression of the *double homeobox protein 4* (*DUX4*) gene in muscle. *DUX4* codes for a transcription factor which, in skeletal muscle, dysregulates numerous signaling activities that culminate in cytotoxicity. Potential treatments for FSHD therefore aim to reduce the expression of *DUX4* or the activity of its toxic protein product. In this article, we review how genetic approaches such as those based on oligonucleotide and genome editing technologies have been developed to achieve these goals. We also outline the challenges these therapies are facing on the road to translation, and discuss possible solutions and future directions

## Introduction

Facioscapulohumeral muscular dystrophy (FSHD) is an autosomal dominant disorder that affects 1 in 8,000–22,000 people in the world and is the third most common inherited muscular dystrophy (Wang and Tawil, 2016). It presents as a progressive, distinctively asymmetric weakening of muscles in the face, shoulders, and upper limbs. Muscles in other regions may become affected with age; around 20% of patients become wheelchair-bound (Richards et al., 2012). Extramuscular symptoms are uncommon, with a few patients experiencing restrictive lung disease, cardiac conduction abnormalities, hearing loss, or retinal vasculopathy (Fitzsimons et al., 1987; Padberg et al., 1995; Laforêt et al., 1998; Lutz et al., 2013; Scully et al., 2014; Lim et al., 2020c). Age of onset and disease severity are both widely variable in FSHD (Tawil et al., 2014; Wang and Tawil, 2016). Intriguingly though, 4–21% of patients who manifest symptoms before the age of five almost all follow a more severe and rapid course of the disease (Klinge et al., 2006; Goselink et al., 2017). There is no available cure for FSHD. Patients are currently managed for their symptoms at best.

While the genetic mechanisms leading to FSHD are diverse and complex, these all result in aberrant expression of the *double homeobox protein 4* (*DUX4*) gene in skeletal muscle. *DUX4* has roles in early embryonic development, where it appears to be essential for zygotic gene activation (De Iaco et al., 2017; Hendrickson et al., 2017). Under healthy conditions, *DUX4* is epigenetically silenced after the 4-cell stage in humans and kept as such in all developed tissues but the testis (Snider et al., 2010) and thymus (Das and Chadwick, 2016). This repression is compromised in patients with FSHD, allowing for the synthesis of the DUX4 transcription factor whose activities in skeletal muscle induce potent cytotoxicity by dysregulating pathways involved in cell death, oxidative stress, and muscle development, among others (Dixit et al., 2007; Lim et al., 2020c).

Various approaches are being explored to treat FSHD. Pharmacological treatments have been evaluated mostly with the aim of improving muscular symptoms, and include the use of prednisone, β2 receptor agonists, myostatin inhibitors, and antioxidants, among others. Unfortunately, these generally offered little to no therapeutic benefit based on results from clinical trials (Hamel and Tawil, 2018; Le Gall et al., 2020). Intramuscular transplantation of myoblasts or mesoangioblasts (perivascular myogenic stem cells) from unaffected muscles of FSHD patients into immunodeficient mice revealed that these could integrate with recipient muscle fibers fairly well (Vilquin et al., 2005; Morosetti et al., 2011). However, follow-up studies examining the benefits of such cell-based therapies on FSHD muscle pathology or function are currently unavailable and so their potential for treating FSHD remains uncertain.

In response to developing a more targeted form of treatment, reducing muscle-specific *DUX4* expression and DUX4-mediated toxicity have become attractive goals for FSHD therapy (Bao et al., 2016; Bouwman et al., 2020; Cohen et al., 2020). Indeed, a number of genetic methods have been employed to achieve one or both of these, including oligonucleotide-based strategies to knockdown *DUX4* transcript levels or reduce DUX4 protein activity, and genome editing to correct FSHD-associated mutations. The pre-clinical development of these strategies and others has shown much promise, and identifies possible candidates for clinical trials. Compared to pharmaceutical and cell-based interventions, genetic treatments target the root cause of the disease (i.e., *DUX4*) and are thus expected to lead to more effective or far-reaching therapeutic effects. In this article, we review the various genetic approaches that have been developed for FSHD therapy, discuss the challenges they may be facing on their way to the clinic, and offer some potential solutions as well as directions for future research.

## *DUX4* Expression and FSHD

**FIGURE 1:**
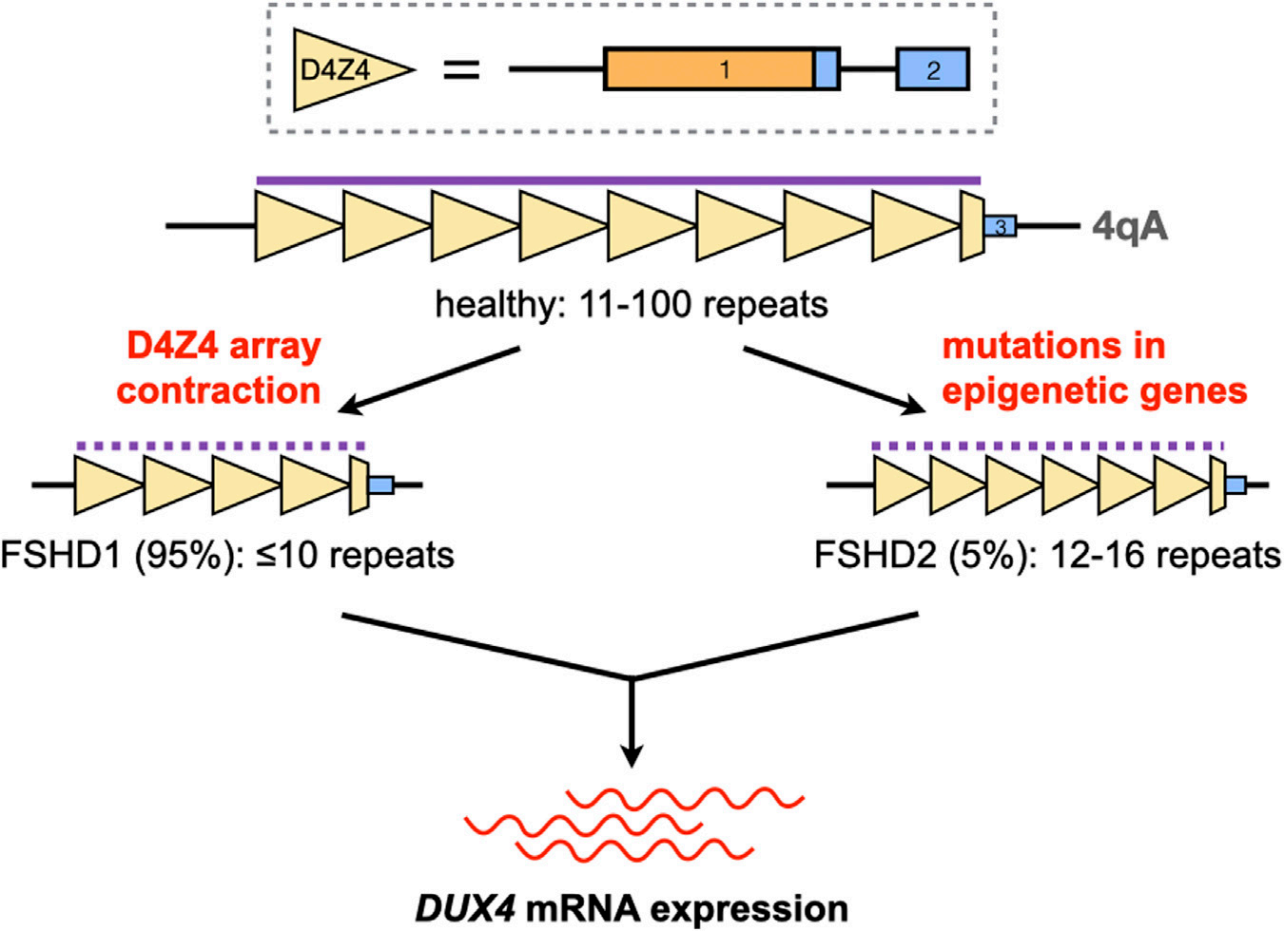
Activation of *DUX4* expression in FSHD. The *DUX4* gene is located in the D4Z4 macrosatellite repeat array at chromosome 4q35. Each D4Z4 repeat (yellow triangles) contains *DUX4* exons 1 and 2 (solid boxes; orange, open reading frame); exon three is found downstream of the last repeat in the array. The D4Z4 array is normally 11–100 repeats long and hypermethylated (purple line) in healthy individuals. Contractions of this array or mutations in genes coding for epigenetic regulators, in the 4qA haplotype, disrupt the silencing of *DUX4* (dotted purple line) and lead to its aberrant expression in skeletal muscle.

Much of the complexity associated with FSHD genetics comes from the curious location of *DUX4* in the genome. The *DUX4* gene is part of the D4Z4 macrosatellite repeat array at chromosome 4q35, which is typically 11–100 repeats long in healthy individuals (Gabriëls et al., 1999; Lemmers et al., 2010). There is a homologous D4Z4 repeat array at chromosome 10q26, but mutations in this region have not been linked to FSHD (Bakker et al., 1995; Deidda et al., 1995; Lemmers et al., 2010). Each D4Z4 repeat contains the first two exons of *DUX4*, with the entire open reading frame of the gene in exon 1 (Gabriëls et al., 1999) **(** Figure 1 **)**. *DUX4* has other exons downstream of the array; the full-length isoform that contributes to FSHD pathology ends at exon 3 (Snider et al., 2010; Himeda and Jones, 2019). Only exons from the last D4Z4 repeat contribute to the *DUX4* mRNA, and a polyadenylation signal (PAS) at exon 3 is required to stabilize the pathogenic *DUX4* transcript, a feature that is only present in the disease-permissive 4qA haplotype (Lemmers et al., 2002; Lemmers et al., 2004; Lemmers et al., 2010). Finally, the 4q35 D4Z4 repeat array is normally hypermethylated, which keeps the *DUX4* gene repressed in most adult tissues (Hewitt et al., 1994). Two mechanisms activate *DUX4* expression in FSHD: D4Z4 repeat array contraction, and mutations in genes coding for epigenetic regulators **(** Figure 1 **)**. These cause approximately 95% (FSHD1) and 5% (FSHD2) of cases, respectively (Wang and Tawil, 2016). Despite vast differences in their underlying genetics, FSHD1 and FSHD2 are clinically indistinguishable, implying that aberrant *DUX4* expression is the key genetic event leading to FSHD pathogenesis.

In FSHD1, contraction of the 4qA D4Z4 array to ≤10 repeats activates *DUX4* expression by increasing chromatin accessibility and promoting DNA hypomethylation in the region (van Overveld et al., 2003; Hewitt, 2015). It was previously thought that individuals with ≤10 D4Z4 repeats in one 4qA chromosome form a homogeneous FSHD1 population, but it is now known that this is not the case. Clinical variability is high in individuals with 7–10 D4Z4 repeats, with most cases ranging from mild to asymptomatic (Ricci et al., 2013; Lemmers et al., 2015). This spread of phenotypes is attributed to inter-individual differences in D4Z4 methylation, indicating that factors other than array contraction may be more important in determining disease penetrance within this repeat range (Van Overveld et al., 2005; Lemmers et al., 2015). Conversely, penetrance is more complete in individuals with 1-6 D4Z4 repeats. Disease severity is also roughly inversely correlated with repeat count in these patients, e.g. those with the severe early-onset form of FSHD typically have 1-3 D4Z4 repeats (Lunt et al., 1995; Ricci et al., 2013; Nikolic et al., 2016). Considering the 1–10 D4Z4 repeat range, it appears that the lower the number of repeats present, the less contribution factors other than contraction size have in influencing the FSHD1 phenotype.

Unlike in FSHD1, moderately-sized D4Z4 arrays are observed in FSHD2. On average, FSHD2 patients have 12–16 D4Z4 repeat units on at least one 4qA chromosome, which is at the shorter end of the range that characterizes the general population (de Greef et al., 2010; Himeda and Jones, 2019). However, at our current level of understanding, D4Z4 array size has little to do with FSHD2 development. Instead, the majority of FSHD2 patients (∼80%) carry mutations in *SMCHD1*, which codes for a protein involved in maintaining repressive chromatin architecture (Lemmers et al., 2012); others have mutations in *DNMT3B* or *LRIF1*, which code for a DNA methyltransferase or an interactor of SMCHD1, respectively (van den Boogaard et al., 2016; Hamanaka et al., 2020). These mutations lead to D4Z4 hypomethylation independent of D4Z4 array size, creating a permissive environment for *DUX4* expression on the 4qA chromosome. One study showed that the extent of D4Z4 hypomethylation correlated with disease severity in FSHD2, at least for *SMCHD1* mutation carriers (Lemmers et al., 2015). Because of their role in D4Z4 methylation, *SMCHD1* and *DNMT3B* are also genetic modifiers for FSHD1, leading to cases with characteristics of both FSHD1 and FSHD2 (Sacconi et al., 2013; van den Boogaard et al., 2016; de Greef et al., 2018; Sacconi et al., 2019).

## Genetic Therapies for FSHD

**FIGURE 2:**
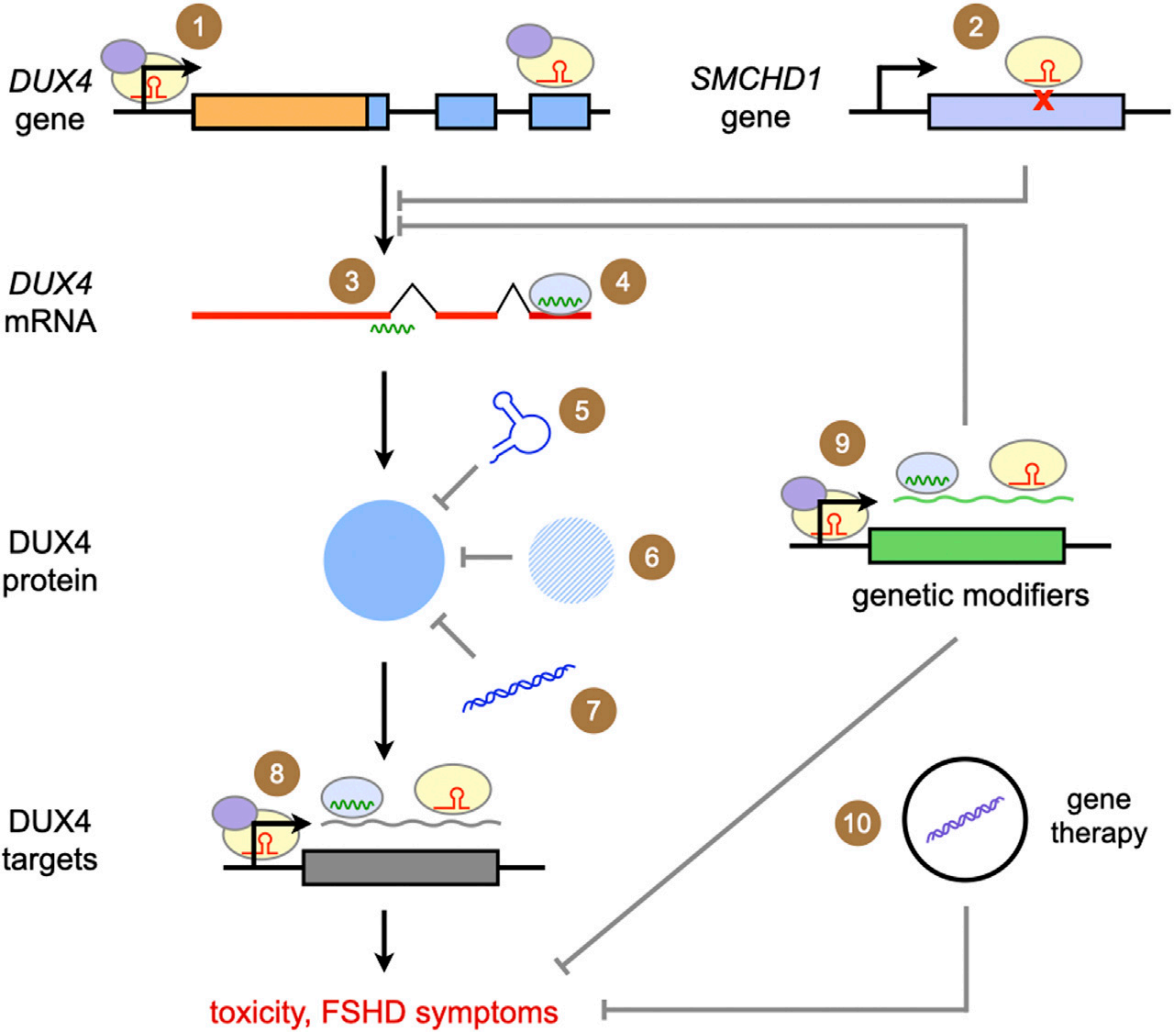
Summary of genetic approaches for the treatment of FSHD. The approaches covered in this review are depicted above. Ultimately, all focus on reducing *DUX4* expression, DUX4 protein activity, or the effects of DUX4-mediated toxicity. (1) Targeted gene repression with CRISPR/dCas9-KRAB; (2) correction of a *SMCHD1* mutation with CRISPR/Cas9; (3) gene knockdown with antisense oligonucleotides; (4) gene knockdown/silencing with RNA interference; competing with DUX4 activity through (5) DNA aptamers, (6) introduction of proteins homologous or similar to DUX4, and (7) DNA decoys; (8) gene knockdown/silencing of DUX4 downstream targets; (9) gene knockdown/silencing of genetic modifiers of *DUX4* expression or DUX4-mediated toxicity (10) delivery of genes coding for proteins that ameliorate DUX4-mediated toxicity or secondary features of FSHD pathology.

Figure 2 summarizes the genetic therapies that have been developed for FSHD, which are covered in the following sections. Briefly, we have potential genetic therapies targeting DUX4 at the DNA, RNA, and protein levels. There are also therapies that focus on inhibiting the effects of DUX4-mediated toxicity, thereby modifying the disease phenotype.

### Oligonucleotide Therapies

Depending on their structure and chemistry, oligonucleotides can inhibit *DUX4* expression in a variety of ways. One of the most extensively tested for FSHD are antisense oligonucleotides (AOs), single-stranded nucleic acid analogues that can bind target mRNA sequences by Watson-Crick base-pairing. There are two kinds of AOs. The first are those that reduce gene expression by interfering with mRNA splicing and processing (Lim and Yokota, 2018). These AOs act as steric blockers, preventing factors from accessing critical sequences in the mRNA such as splice sites, and are usually phosphorodiamidate morpholino oligomers (PMOs) or phosphorothioated 2′-O-methyl RNAs (2′-OMePS). The second are those that reduce gene expression by inducing target mRNA degradation (Lim and Yokota, 2020). The AOs in this group are gapmers, fully phosphorothioated oligonucleotides that have a central DNA stretch flanked by bases of modified chemistry, e.g. locked nucleic acids (LNA) or 2′-O-methoxyethyl RNAs (2′-MOE). When a gapmer binds its target mRNA, a DNA/RNA hybrid is created in the middle of the AO that is recognized by ribonuclease H, which proceeds to bind the hybrid and cleave its RNA portion.

**FIGURE 3:**
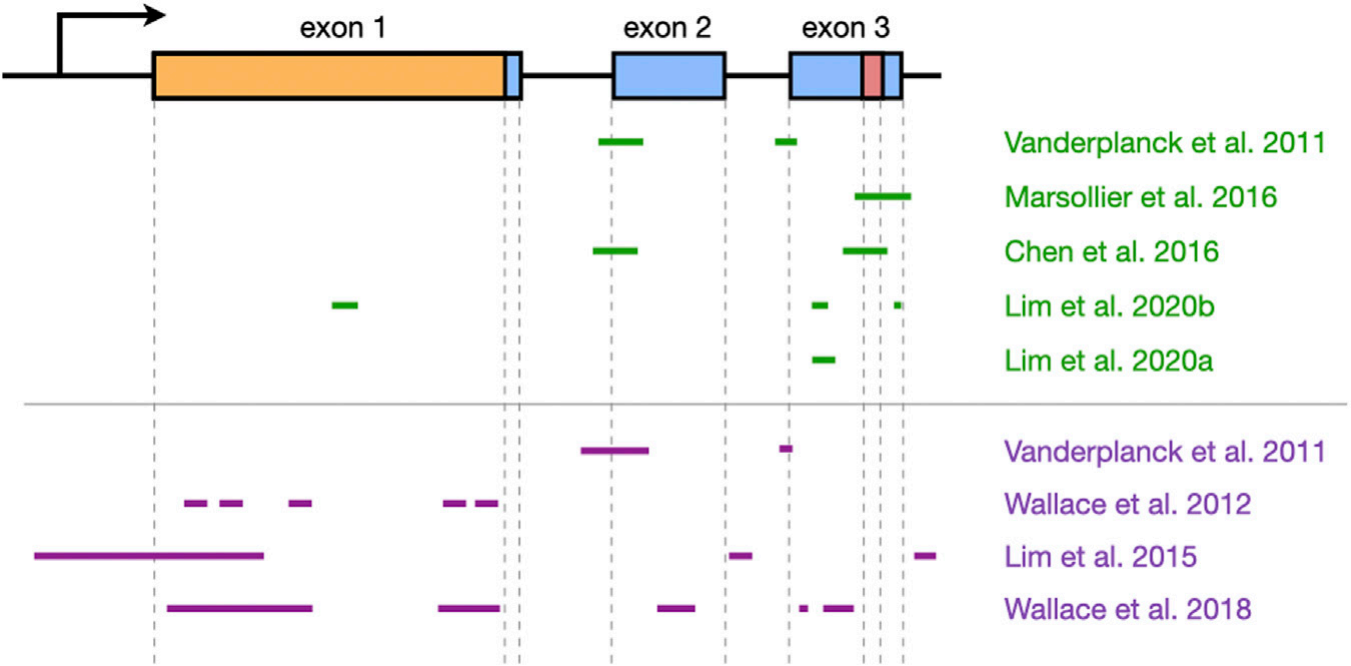
Overview of *DUX4* regions that have been targeted by oligonucleotide therapies. The structure of the *DUX4* gene is shown at the top (arrow indicates promoter region; boxes, exons; lines, introns; orange, open reading frame; red, polyadenylation signal), and the regions that have been targeted by antisense oligonucleotides (green) or RNA interference (purple) are shown at the bottom. Approximate locations are shown, and the figure is not to scale. Note that Ansseau et al. (2017) used the same oligonucleotides as Vanderplanck et al. (2011). *DUX4* structure was based on information from Ensembl, transcript ID ENST00000569241.5.

AOs of both kinds have successfully inhibited *DUX4* expression in patient-derived cells and FSHD mouse models **(** Table 1 **)**. PMOs and 2′-OMePS AOs targeting splice acceptor sites for *DUX4* exons 2 and 3 **(** Figure 3 **)** gave 30–90% *DUX4* mRNA knockdown (at 10 and 50 nM tested doses) in myotubes from treated primary patient myoblasts (Vanderplanck et al., 2011; Ansseau et al., 2017). Corresponding reductions in DUX4 downstream target gene expression and DUX4-positive nuclei, as well as improvements in muscle cell morphology, were observed. AOs targeting the exon 3 splice acceptor site were particularly more effective, one of which was tested in mice as a vivo-PMO (Ansseau et al., 2017; Derenne et al., 2020). Vivo-PMOs are PMOs that have been covalently linked to an octaguanidine dendrimer for improving uptake in tissues (Morcos et al., 2008). Mice transduced with *DUX4* constructs at the tibialis anterior (TA) were intramuscularly (i.m.) injected at the same muscle with 10 μg of the vivo-PMO, which led to 30-fold lower *DUX4* expression than the control vivo-PMO-treated leg by semi-quantitative RT-PCR, 10 days after treatment (Ansseau et al., 2017). Histopathological improvements were observed in another study using the same AO (Derenne et al., 2020). PMOs have also been used to target the PAS in exon 3 **(** Figure 3 **)**, which knocked down *DUX4* transcript expression in immortalized patient-derived myotubes by 25–52% at a 50 nM dose (Marsollier et al., 2016) and in a xenograft FSHD mouse model by nearly 100% with a 20-μg injection (Chen et al., 2016a). Reduced expression of DUX4 downstream target genes, transcriptomic-level restoration, and loss of DUX4-positive nuclei were observed *in vitro*; treatment showed no significant improvements in muscle cell fusion, however.

**TABLE 1:** Summary of results from pre-clinical studies on antisense oligonucleotides for *DUX4* knockdown.

Study	Chemistry	*DUX4* target	Model	*DUX4* knockdown (dose)	Other results
Vanderplanck et al. (2011)	2′-OMePS	Ex2 SA, Ex3 SA	Primary FSHD myoblasts, differentiated post-treatment	30% (ex2 SA, 50 nM), 50% (ex3 SA, 10 nM)	Reduced TP53 levels, *TRIM43* expression
Marsollier et al. (2016)	PMO	Ex3 PAS, down-stream elements	Immortalized FSHD myotubes	25–52% (50 nM)	Reduced DUX4 downstream gene expression; fusion not affected
Chen et al. (2016a)	PMO	Ex2 SA, Ex3 PAS	Primary FSHD myotubes	Not assessed	Reduced DUX4^+^ nuclei, DUX4 downstream gene expression (only for PAS PMOs); transcriptomic improvements
		Ex3 PAS	FSHD xenograft mice, 1x e.p. into xenograft, evaluated 2 weeks post-treatment	∼100% (20 μg)	Reduced DUX4 downstream gene expression
Ansseau et al. (2017)	2′-OMePS	Ex2 SA, Ex3 SA	Primary aFSHD and dFSHD myoblasts, differentiated post-treatment	∼90% (ex2 SA, 50 nM; ex3 SA, 10 nM)	Reduced DUX4^+^ nuclei; saw improvements in size (in aFSHD but not dFSHD myotubes)
	Vivo-PMO	Ex3 SA	AAV-DUX4 mice, 1x i.m. TA, evaluated 10 days post-treatment	30-Fold lower than control vivo-PMO	None
Derenne et al. (2020)	Vivo-PMO	Ex3 SA	DUX4 IMEP mice, 1x i.p., evaluated 1 week post-treatment	Not assessed	2.5-fold decrease in histological lesion compared to non-treated
Lim et al. (2020b)	LNA gapmer	Ex1, Ex3	Immortalized FSHD myotubes	∼100% (100 nM)	Reduced DUX4 downstream gene expression; partial transcriptomic restoration; improved muscle cell fusion/size
		Ex3	*FLExDUX4* mice, 3x i.m., evaluated 1 or 7 days post-treatment	84% (1 day, 20 μg/i.m.), 70% (7 days, 20 μg/i.m.)	Gapmer uptake observed in and between muscle fibers
Lim et al. (2020a)	2′-MOE gapmer	Ex3	Immortalized FSHD myotubes	∼100% (100 nM)	Reduced DUX4 downstream gene expression; partial transcriptomic restoration; improved muscle cell fusion/size
		Ex3	*FLExDUX4* mice, 3x i.m., evaluated 1 day post-treatment	∼65% (20 μg/i.m.)	None

2′-OMePS, phosphorothioated 2′-O-methyl RNAs; PMO, phosphorodiamidate morpholino oligomer; LNA, locked nucleic acid; 2′-MOE, 2′-O-methoxyethyl; Ex, exon; SA, splice acceptor; PAS, polyadenylation signal; e.p., electroporation; i.m., intramuscular injection; i.p., intraperitoneal injection; TA, tibialis anterior; AAV, adeno-associated virus; IMEP, intramuscular injection and electroporation of naked plasmid DNA; aFSHD, atrophic FSHD myotubes; dFSHD, disorganized FSHD myotubes.

Our group has recently published on the efficacy of using gapmers for inhibiting *DUX4* mRNA expression. We designed LNA gapmers to target sites at *DUX4* exons 1 and 3 (Lim et al., 2020b), as well as 2′-MOE gapmers to target only sites at exon 3 (Lim et al., 2020a) **(** Figure 3 **)**. The LNA and 2′-MOE gapmers targeted overlapping sequences at exon 3, upstream of the PAS. All gapmers knocked down *DUX4* mRNA levels almost completely (∼99%) at 100 nM and by more than 50% at 10 nM regardless of chemistry in immortalized patient-derived myotubes. This demonstrates an increased potency of gapmers compared to steric-blocking AOs, perhaps due to the direct nature of transcript degradation induced by the gapmers. More sustained knockdown of DUX4 downstream target genes was observed for LNA than 2′-MOE gapmers at 10 nM. LNA gapmer treatment also restored more FSHD signature genes upon RNA sequencing analysis, hinting that LNA gapmers may be the more potent of the two in terms of *DUX4* knockdown. Focusing on exon 3-targeting gapmers, we saw improvements in muscle cell fusion and size, as well as minimal to no effects on potential off-target genes *in vitro*. A separate study also found that treatment with one of the 2′-MOE gapmers increased membrane repair in immortalized patient myoblasts (Bittel et al., 2020). One gapmer from each chemistry was also tested in the *FLExDUX4* model, which carry a stably integrated, Cre-inducible *DUX4* transgene (Jones and Jones, 2018). Non-induced *FLExDUX4* mice exhibit low levels of *DUX4* expression mimicking what is seen in patient muscle cells, and was used for preliminary studies. Significant *DUX4* knockdown was induced in these mice following three 20-μg i.m. TA injections, at 70–84% for the LNA gapmer and 65% for the 2′-MOE gapmer, on average.

Another class of oligonucleotide therapy is RNA interference (RNAi), which makes use of small interfering RNAs (siRNAs) or microRNAs (miRNAs) **(** Table 2 **)**. Unlike AOs, siRNAs and miRNAs require association with effector proteins to reduce target gene expression. siRNAs targeting *DUX4* promoter elements or exons **(** Figure 3 **)** knocked down *DUX4* transcript levels by 50–90% *in vitro*, with corresponding restorative effects on DUX4 downstream targets (Vanderplanck et al., 2011; Lim et al., 2015). Interestingly, siRNAs against the promoter likely inhibited *DUX4* expression through epigenetic silencing at the DNA level, since 2′-MOE gapmers against the same region did not affect *DUX4* transcript levels (Holoch and Moazed, 2015; Lim et al., 2015). Meanwhile, one group screened a large number of miRNAs **(** Figure 3 **)** and found two targeting exon 1 (mi1155, mi405) to knock down *DUX4* expression the best at >75% in *DUX4*-luciferase reporter cells (Wallace et al., 2012; Wallace et al., 2018). Treatment of *DUX4*-transduced mice (i.m., TA) with 3 × 10^10^ adeno-associated viruses (AAVs) carrying mi405 constructs reduced *DUX4* mRNA expression by 64%, and DUX4 protein levels by 90% (Wallace et al., 2012). Histopathology was improved with this miRNA, but not with mi1155 that instead showed signs of overt toxicity (Wallace et al., 2018).

**TABLE 2:** Summary of results from pre-clinical studies on RNA interference for *DUX4* knockdown.

Study	Approach	*DUX4* target	Model	*DUX4* knockdown (dose)	Other results
Vanderplanck et al. (2011)	siRNA	Ex2 SA, Ex3 SA	Primary FSHD myoblasts, differentiated post-treatment	80% (ex3 SA, 10 nM)	Reduced DUX4, Atrogin1, TP53, protein levels, and MuRF1^+^ nuclei; improved muscle size
Wallace et al. (2012)	miRNA	Ex1	DUX4-luciferase reporter HEK293 cells	>50% (dose not given)	Reduced DUX4 protein levels
		Ex1	AAV-DUX4 mice, 1x i.m. TA, evaluated 2–4 weeks post-treatment	64% (3 × 10^10^ particles)	Reduced DUX4 protein levels (90%); improved histopathology; lack of caspase-3^+^ myofibers; improved grip strength
Lim et al. (2015)	siRNA	Promoter, Ex1, In2, downstream elements	Primary FSHD myoblasts, differentiated post-treatment	Up to ∼50–90% (100 pmol)	Reduced DUX4^+^ nuclei, *ZSCAN4* expression
Wallace et al. (2018)	miRNA	Ex1, Ex2, Ex3	DUX4-luciferase reporter HEK293 cells	Up to >75% (dose not given)	Reduced DUX4 protein levels (up to >75%)
		Ex1	AAV-DUX4 mice, 1x i.m. TA/isolated limb perfusion, evaluated at various timepoints post-treatment	Not assessed	One miRNA was more toxic than the other upon histological evaluation

siRNA, small interfering RNA; miRNA, microRNA; Ex, exon; In, intron; SA, splice acceptor; i.m., intramuscular injection; TA, tibialis anterior; AAV, adeno-associated virus.

Oligonucleotides can also be designed to target the DUX4 protein. Double-stranded DNA decoys containing the DUX4 binding motif have recently been developed to sequester and prevent DUX4 from activating its downstream targets (Mariot et al., 2020). Indeed, the expression levels of DUX4 downstream targets *ZSCAN4* and *TRIM43* were knocked down by 39–91% in primary patient myotubes upon treatment with these decoys. The DNA decoys were also tested in AAV-DUX4 mice, where administration either by intramuscular electroporation or AAV delivery led to decreased expression of *Tm7sf4*, another DUX4 downstream target. On a related note, single-stranded DNA aptamers have recently been developed with high, preferential affinity to the DUX4 DNA-binding domain (Klingler et al., 2020). However, these aptamers have yet to be tested for their therapeutic potential. Developing oligonucleotides for targets other than *DUX4* may be useful as well. For instance, *PITX1* is a direct transcriptional target of DUX4 whose overexpression induces an FSHD-like dystrophic phenotype in mice (Dixit et al., 2007; Pandey et al., 2012). Intravenous injection of AOs against *Pitx1* in *Pitx1*-transgenic mice improved grip strength and decreased muscle pathology (Pandey et al., 2014). *FRG1* is another direct transcriptional target of *DUX4*, whose knockdown by RNAi reversed dystrophic histopathology and improved treadmill performance in *FRG1*-overexpressing mice (Bortolanza et al., 2011; Ferri et al., 2015). It would be interesting to see if similar effects could be observed if these strategies were used to treat *DUX4*-overexpressing mouse models such as *FLExDUX4* (Jones and Jones, 2018), the doxycycline-inducible iDUX4pA (Bosnakovski et al., 2017a; Bosnakovski et al., 2020), or the tamoxifen-inducible TIC-DUX4 (Giesige et al., 2018).

### CRISPR

The bacterial defense system based on clustered regularly interspaced short palindromic repeats (CRISPR) has been adapted and developed to become perhaps one of the most revolutionary tools for targeted genome editing to date. In its most common configuration, CRISPR has two basic components: an endonuclease for cleaving DNA (the CRISPR-associated or Cas protein), and an RNA molecule that associates with this enzyme and tells it where in the genome to cut (the guide RNA or gRNA) (Jinek et al., 2012; Jiang and Doudna, 2017). The gRNA is designed complementary to the target DNA site, which additionally has to have a protospacer-adjacent motif sequence nearby to facilitate Cas binding (Mojica et al., 2009; O’Connell et al., 2014). Upon binding of the gRNA-Cas complex, a double-stranded break is introduced into the target DNA. This break is subsequently resolved by non-homologous end joining or homology-directed repair, which create random insertions/deletions or precise edits at the site, respectively, and form the basis of CRISPR-based genome editing.

CRISPR has been previously used to correct an FSHD2-associated *SMCHD1* mutation, a missense variant in intron 34 that introduced an out-of-frame 53-bp pseudoexon in the final transcript (Goossens et al., 2019). CRISPR/Cas9 with gRNAs against the intronic sequences flanking this pseudoexon restored the *SMCHD1* reading frame and increased wild-type *SMCHD1* expression in primary and immortalized patient myotubes, resulting in reduced *DUX4* mRNA expression. It has been suggested that CRISPR be used to edit the permissive 4qA to the restrictive 4qB haplotype (Cohen et al., 2020), but attempts on realizing this approach have not yet been reported in the literature. In addition to genome editing, CRISPR can also be used for the targeted modulation of gene expression. Using a catalytically-deficient version of Cas9 (dCas9) fused to a KRAB transcriptional repressor, together with gRNAs against the *DUX4* promoter or exon 1, one group achieved ∼45% *DUX4* knockdown in myotubes differentiated from treated primary patient myoblasts (Himeda et al., 2016). A trend toward increased chromatin repression of the *DUX4* gene at the contracted locus was observed. When dCas9-KRAB was used with gRNAs solely targeting *DUX4* exon 3 or various regions within/upstream of the D4Z4 repeat sequence, no significant *DUX4* knockdown was observed. The same group used dCas9-KRAB to inhibit the expression of other genes—*BRD2*, *BAZ1A*, *KDM4C*, and *SMARCA5*—which led to about 40–60% *DUX4* knockdown in primary patient myotubes (Himeda et al., 2018). These genes code for epigenetic regulators, and were previously identified from an RNAi screen as candidates whose knockdown lowered *DUX4* transcript levels without negatively impacting the expression of genes involved in muscle development or homeostasis. On a related note, CRISPR/Cas9 has itself been employed for a genome-wide knockout screen to search for genes whose loss-of-function was protective against DUX4 cytotoxicity (Lek et al., 2020). Hypoxia signaling pathway members were identified as the most promising candidates, in accordance with the role of oxidative stress in DUX4-mediated pathogenesis (Dmitriev et al., 2016; Denny and Heather, 2017; Lim et al., 2020c).

### Other Approaches

Preliminary findings from basic research studies are providing solid foundations for the development of more strategies for FSHD therapy. One interesting approach is to use other proteins to compete with DUX4 activity. DUX4-s is a short isoform of DUX4 that contains only the first 159 N-terminal amino acids of the protein, spanning both homeodomains (Mitsuhashi et al., 2018). It is non-pathogenic, and its expression has been detected in both healthy and FSHD skeletal muscle (Snider et al., 2010; Geng et al., 2011; Mitsuhashi et al., 2018). Since DUX4-s shares the exact same homeodomains as full-length DUX4, it is thought that overexpression of the former will prevent the latter from binding its usual genomic targets. Indeed, co-injection of *DUX4-s* and full-length *DUX4* mRNA at a 20:1 ratio into fertilized zebrafish eggs decreased embryo mortality rates to ∼10%, improved musculature, and led to 70% of embryos having an overall normal phenotype (Mitsuhashi et al., 2013). In contrast, eggs injected with only full-length *DUX4* mRNA had an embryo mortality rate of ∼40%, and less than 20% of resulting embryos were phenotypically wild-type. As the physiological functions of DUX4-s are unknown, more research into this area may help further develop this approach as an FSHD therapy. The DUX4 homeodomains are also highly similar and functionally interchangeable with those of PAX7 (Bosnakovski et al., 2017b). Overexpression of *Pax7* or its homolog *Pax3* considerably improved viability in *DUX4*-inducible C2C12 cells (Bosnakovski et al., 2008). This rescue was diminished in a dose-dependent manner when *DUX4* expression was induced at higher levels, indicating that Pax7 or Pax3 may be exerting their effects via competition with the DUX4 protein. Although promising, pre-clinical testing of DUX4-s and PAX7/3 in FSHD mouse models have yet to be performed.

Research into understanding FSHD biology or *DUX4*-mediated cytotoxicity has also uncovered more potential targets for therapy. These include genes involved in apoptosis (*CDKN1A*, *MYC*), immune response activation (*RNASEL*, *EIF2AK2*), and epigenetic regulation (*H3.X*, *H3.Y*), to name a few (Lim et al., 2020c). As previously mentioned, RNAi and CRISPR screens have been instrumental in adding to this list by identifying genetic modifiers of *DUX4* expression (Himeda et al., 2018; Lek et al., 2020). Modulating the expression of these genes, either by oligonucleotide- or CRISPR-based approaches, may be therapeutic avenues worth investigating. Developing treatments to alleviate FSHD symptoms may be beneficial as well. For instance, AAV delivery of a follistatin gene construct into TIC-DUX4 FSHD model mice (i.m.) increased mass and improved strength in injected muscles (Giesige et al., 2018). Follistatin is an inhibitor of myostatin, which in turn is a known inhibitor of muscle growth (Rodino-Klapac et al., 2009). It is important to note though that follistatin did not reverse DUX4-induced histopathology in treated mice, suggesting that treatments directed at secondary pathological features of FSHD are probably not curative and may be more useful when administered in conjunction with *DUX4*-targeting genetic therapies.

## Challenges for FSHD Genetic Therapies

The development of genetic therapies for FSHD is at a relatively young phase, with most pre-clinical work limited to *in vitro* models. Only a handful of these experimental therapies have moved on to *in vivo* testing **(** Table 1 **)**, of which only one was evaluated for its efficacy in ameliorating FSHD symptoms (Wallace et al., 2012; Wallace et al., 2018). This may be explained by the lack of appropriate FSHD animal models at the time, an effort that was largely hindered by the lethal effects of DUX4 (DeSimone et al., 2020). Various groups have since capitalized on using conditional methods to overexpress *DUX4* in mice, allowing for more refined control of DUX4 toxicity and ushering in the production of FSHD animal models amenable to pre-clinical study (Bosnakovski et al., 2017a; Bosnakovski et al., 2020; Giesige et al., 2018; Jones and Jones, 2018). With the impending progression of FSHD genetic therapies into *in vivo* testing, certain challenges will have to be considered and overcome to ensure treatment success not only in animal models but also and ultimately in patients. We consider challenges relevant to oligonucleotide- and CRISPR-based therapies, as these have advanced the most in pre-clinical development.

Delivery is perhaps one of the largest hurdles these therapies have to face *in vivo*. In order for genetic therapies to work, they will have to reach their target DNA/RNA sequences in the nuclei of their target cells. Preliminary work using AOs for instance have shown that levels of *DUX4* knockdown *in vivo* are not usually as high as those observed *in vitro* (Wallace et al., 2012; Ansseau et al., 2017; Lim et al., 2020a; Lim et al., 2020b). The same divide *in vitro* and *in vivo* efficacy has been observed for similar genetic approaches in other muscular disorders, such as Duchenne muscular dystrophy (DMD) (Echigoya et al., 2017; Lim et al., 2019; Nguyen and Yokota, 2019). For oligonucleotide therapies, much of the challenge will be to get them into muscle cells and, once inside, to have them successfully escape from endosomes and reach the nucleus (Juliano, 2016). This is especially problematic for charge-neutral chemistries such as PMOs (Summerton and Weller, 1997), which are not readily recognized by cell surface receptors nor are they particularly disruptive toward cell membranes. Conjugation of cell-penetrating entities to oligonucleotides in order to enhance cellular uptake and endosomal escape are actively being investigated, as are the use of non-viral delivery vehicles like lipid nanoparticles, polymeric nanocarriers, and exosomes (Dominska and Dykxhoorn, 2010; Juliano, 2016; Tong et al., 2019). Chemically modifying the oligonucleotides themselves to appear more recognizable to muscle cell surface receptors is currently being looked into as well (Dominska and Dykxhoorn, 2010; Juliano, 2016; Tong et al., 2019). Viral vectors may be applicable for RNAi strategies using miRNAs or shRNAs, but their immunogenicity will have to be carefully considered when delivered *in vivo*.

CRISPR components, on the other hand, have mostly been delivered using viral vectors. This has mostly been effective, except for two major concerns: the packaging limit into viruses and immunogenicity. The SpCas9 (the most used Cas variant) gene construct is ∼4.2 kb long, which is quite near the packaging capacity of AAVs at ∼4.4–4.7 kb (Wu et al., 2010; Tong et al., 2019; Wilbie et al., 2019). Use of a two-vector delivery system has been necessary at times, one for Cas9 and another for the gRNA/s. How this division exactly affects CRISPR efficacy is still poorly understood, but could be unfavorable when considering that we have to maximize the likelihood of all CRISPR components being present in the same nuclei—a challenge that is perhaps made more difficult by the syncytial nature of mature muscle cells and the fact that only a very few nuclei actually express *DUX4* (Tassin et al., 2013). Lentiviral and retroviral vectors have larger capacities at 7–10 kb, but are prone to inducing unwanted integration of their cargo into the host genome (Tong et al., 2019). Adenoviral vectors have even larger capacities at about 36 kb, and are emerging as effective vehicles for CRISPR delivery (Ehrke-Schulz et al., 2017; Tong et al., 2019; Ricobaraza et al., 2020). The discovery of smaller Cas variants such as CjCas9 (2.95 kb gene size) is also helping overcome this packaging limit (Kim et al., 2017). However, due to their immunogenicity, there is always the risk of complications and so the use of immunosuppressive agents remain important, especially since responses may still be activated at high doses of virus, and individual reactions to viral agents are difficult to predict (Tong et al., 2019; Xu et al., 2019; Shirley et al., 2020). Non-viral delivery approaches, similar to those described for oligonucleotide therapies, are also being developed for CRISPR to overcome both issues of packaging and immunogenicity (Lino et al., 2018; Wilbie et al., 2019).

Safety is another concern. Oligonucleotide and CRISPR therapies can be toxic through hybridization-dependent or -independent means (Frazier, 2015). One aspect of hybridization-dependent toxicity is off-target gene knockdown/knockout/editing, resulting from the recognition of non-target sequences that share imperfect complementarity with a given oligonucleotide or gRNA. Only a few studies on *DUX4*-targeting therapies have examined off-target effects—dose-dependent knockdown was found in some non-target genes, whereas no effect was observed in others (Lim et al., 2020a; Lim et al., 2020b). A more thorough analysis of off-target effects is recommended for future studies, perhaps using genome- or transcriptome-wide approaches (Kim et al., 2015; Tsai et al., 2015; Yoshida et al., 2019). Fortunately, the specificity of these genetic therapies is constantly being improved through chemical modification or by performing *in silico* screens to predict the off-targeting potential of certain oligonucleotide sequences prior and in addition to *in vitro* testing (Kamola et al., 2015; Tycko et al., 2016; Hendling and Barišić, 2019; Wang et al., 2019). In the case of CRISPR, splitting the Cas enzyme into two interdependent halves and limiting the duration of Cas activity via self-restricting mechanisms have emerged as possible approaches to reduce the chance of off-target effects (Ran et al., 2013; Shen et al., 2014; Moore et al., 2015; Chen et al., 2016b).

Hybridization-dependent toxicity can also come from on-target effects. This is not much of an issue for the FSHD-associated *DUX4* isoform, since it is not supposed to be expressed in the first place, and its expression appears to be muscle-specific (Himeda et al., 2014). Non-pathogenic *DUX4* isoforms are, however, expressed in healthy tissues (Snider et al., 2010; Himeda and Jones, 2019). As the physiological functions of these isoforms are currently unknown, it would be hard to predict the consequences of their reduced expression. Regardless, as a form of caution, genetic therapies should be designed to preferentially target skeletal muscle or regions specific to the pathogenic *DUX4* isoform. Use of viruses with muscle-specific tropism or ligand-directed oligonucleotides/non-viral delivery vehicles can help ensure tissue-specific treatment (Juliano, 2016). As for target sequence design, exon 3 is the region most specific to the pathogenic, full-length *DUX4* isoform. Most oligonucleotide therapies target this exon and so are not particularly concerning; however, therapies targeting other exons, the *DUX4* promoter region, or D4Z4 sequences on other tissues will have to be closely monitored for potential adverse effects. Another on-target effect is the potential integration of genetic material from viral delivery vectors—however, this threat can be minimized by using AAVs or non-viral delivery methods.

On the other hand, hybridization-independent toxicity refers to effects caused by the therapies themselves that are not attributed to their intended sequence-dependent genetic activities. Certain secondary structures on oligonucleotides and gRNAs can be recognized by pattern recognition receptors and lead to an innate immune response (Agrawal and Kandimalla, 2004; Kim et al., 2004; Lee and Yokota, 2013; Kim et al., 2018; Wienert et al., 2018; Wilbie et al., 2019). The formation of such structures therefore has to be considered during the sequence design process for these modalities. Pre-existing adaptive immunity against Cas proteins is also common in the population (Chew, 2018; Crudele and Chamberlain, 2018; Wagner et al., 2019). Whereas Cas proteins are now being engineered to become less immunogenic (Ferdosi et al., 2019; Mehta and Merkel, 2020), efforts to reduce the persistence of Cas activity as described previously are also potential solutions. Aside from immune response activation, hepatotoxicity and nephrotoxicity have been previously linked to phosphorothioated AOs (Frazier, 2015). This toxicity has been partially attributed to the propensity of AOs to bind cellular proteins (Brown et al., 1994; Liang et al., 2015; Kakiuchi-Kiyota et al., 2016; Shen et al., 2018). Accordingly in one study, chemical modifications that reduced the overall protein-binding affinity of gapmers prevented hepatotoxicity in mice, without sacrificing therapeutic efficacy (Shen et al., 2019). Certain sequence patterns have also been found to correlate with the hepatotoxic potential of oligonucleotides (Hagedorn et al., 2013; Burdick et al., 2014). The development of predictive *in silico* tools may help prevent the integration of such sequences during oligonucleotide design (Hagedorn et al., 2013); use of *in vitro* screening for potential AO toxicity prior to therapeutic evaluation would be recommended (Dieckmann et al., 2018; Shen et al., 2019).

Another challenge to consider is that *DUX4* expression is very rare in skeletal muscle, only detected in 1/1000 or 1/200 nuclei in FSHD myoblasts and myotubes, respectively (Snider et al., 2010; Tassin et al., 2013). This poses an issue when evaluating the efficacy of *DUX4*-targeting therapies, by preventing a robust read-out of *DUX4* knockdown. This explains why some studies have instead opted to use *DUX4*-transfected models, which may create an overly toxic, non-representative environment compared to what is seen in patients. Fortunately, we now have robust protocols that enable reliable detection of endogenous *DUX4* mRNA levels, based on real-time quantitative reverse transcription PCR or RNA *in situ* hybridization (Lim et al., 2020b, Lim et al., 2015; Amini Chermahini et al., 2019). Induction of endogenous *DUX4* expression using media supplements such as KOSR has been likewise helpful (Pandey et al., 2015). DUX4 protein detection has shown some success, owing to the development of good antibodies (Dixit et al., 2007; Snider et al., 2010; Geng et al., 2011), but may remain a challenge in certain conditions when considering the potentially short half-life of the DUX4 protein (Geng et al., 2011; Rickard et al., 2015) or the proposed stochastic model of *DUX4* expression in muscle cells (Snider et al., 2010; Rickard et al., 2015). Of course, evaluating effects on DUX4 downstream transcriptional targets or FSHD biomarkers as proxies for *DUX4* knockdown remain options for therapeutic evaluation.

Finally, one has to recognize the unique clinical presentation of FSHD when translating these genetic therapies into patients. One of the most distinctive features of FSHD is its asymmetric phenotype (Wang and Tawil, 2016). We are far from understanding how this occurs—what determines which parts of the muscle get affected first, why certain muscle groups are spared more than others, why the pattern of muscle weakness differs between patients, and so on. The best strategy at the moment would probably be to develop an approach that equally benefits all muscle groups. This requires that therapeutic efficacy be evaluated in representative muscles across the body during *in vivo* testing, and not only on commonly assessed muscles such as the TA. If anything, therapeutic effects on muscles in the upper parts of the body have to be prioritized, given their early involvement in FSHD (Wang and Tawil, 2016). Another approach would be to administer genetic therapies locally, injecting only the affected muscles. While this takes advantage of the patchy nature of FSHD pathology, this method requires a way to reliably locate affected muscle areas and may not be practical if too many muscles have become involved; it will also not improve the state of any extramuscular symptoms. Needless to say, developing therapies that would address the unique characteristics of FSHD would require the use of animal models that resemble the disease fairly well. As previously mentioned, recent advancements in the conditional control of *DUX4* expression have helped us get closer to generating such models, resulting in mice with dystrophic histopathology, impaired muscle strength, and asymmetric muscle degeneration similar to those seen in patients (DeSimone et al., 2020). Due to the inducible nature of *DUX4* expression in these models, the severity of resulting phenotypes is also tunable, allowing for therapeutic testing in a variety of disease states. This is a rather helpful aspect, considering the wide variability in disease presentation seen in patients with FSHD.

## Conclusion

The discovery of *DUX4* as the genetic cause of FSHD has greatly accelerated efforts not only to understand the disorder but also to treat it. As we have seen, *DUX4* has proven itself to be a useful target for genetic therapy. Oligonucleotide- and CRISPR-based approaches have demonstrated the feasibility of *DUX4* knockdown in reversing muscle-specific FSHD pathology, with promising preliminary results *in vitro* and *in vivo*. However, there is much to be done before these therapies can reach the patients they are meant for. Challenges in delivery, efficacy, safety, and in dealing with the unique pathology of FSHD all have to be taken into consideration *in vivo*. The field can use the lessons learned from the application of these therapies in other muscular disorders to help overcome such hurdles. Development of alternative genetic approaches for FSHD therapy should also be encouraged. For instance, strategies to interfere with DUX4 transcription factor activity or to modulate the expression of genes that impart protection against the cytotoxicity of DUX4 appear to have therapeutic potential. The ongoing identification of genes involved in FSHD pathogenesis by basic research and transcriptomic studies are providing new targets for genetic therapies as well. Finally, although FSHD is predominantly a skeletal muscle disease, it will be necessary to create therapies that address its extramuscular symptoms, especially since these tend to manifest in the more severe cases of the disease. Further investigations into both the pathological and physiological roles of *DUX4* in other tissues are steps toward achieving this goal. Such studies should also help us better assess the possible effects of *DUX4*-targeting genetic therapies in non-muscle tissues. Appreciating all the work that has been accomplished thus far, we have certainly come a long way in the development of genetic therapies for FSHD. With continued efforts from both basic and translational research teams in the FSHD community, it may only be a matter of time until we see these therapies making their way into clinical trials.
